# Does children’s healthcare seeking change after participation in a musculoskeletal study? A register-based study

**DOI:** 10.1186/s12875-023-02233-z

**Published:** 2023-12-13

**Authors:** Charlotte Raadkjaer Lykkegaard, Niels Wedderkopp, Sonja Wehberg, Sinead Holden, Helene Stoettrup Andersen, Frans Boch Waldorff, Jens Søndergaard

**Affiliations:** 1 The Research Unit of General Practice, Department of Public Health, University of Southern Denmark, DK-5230 Odense, Denmark; 2 Center for Research in Childhood Health, Department of Regional Health Research, University of Southern Denmark, DK-5230 Odense, Denmark; 3 Centre for General Practice at Aalborg University, Aalborg University, 9220 Aalborg East, Denmark; 4 Department of Health Science and Technology, Aalborg University, 9220 Aalborg East, Denmark; 5 UCD Clinical Research Centre, School of Medicine, University College Dublin, Dublin 4, Ireland; 6 Section of General Practice and The Research Unit for General Practice, Department of Public Health, University of Copenhagen, 1353 Copenhagen, Denmark

**Keywords:** Primary care, Healthcare usage, Children, Register-based, Injury surveillance musculoskeletal complaints, Healthcare seeking behaviour

## Abstract

**Background:**

Participating in research studies often involves interactions with healthcare professionals, potentially influencing the participant’s future help-seeking behaviour. We investigated whether participating in the Childhood Health Activity and Motor Performance School Study – Denmark (CHAMPS) (2008–2014), which involved telephone consultations and clinical assessments by healthcare professionals with participants experiencing musculoskeletal complaints, changed frequency of contacts with primary public healthcare services among participants over the subsequent five-years-period, compared to non-participating children.

**Methods:**

Using Danish health register data from 1998 to 2020, we compared CHAMPS participant’s and two control group’s contacts with private physiotherapists, chiropractors (outside hospitals), and general practitioners: a random 10% sample of children from Denmark (National Controls), and a secondary local control group (Local Controls) during three periods: Before (1998–31.10.2008), during (01.11.2008–20.06.2014), and after (21.06.2014–31.12.2019) the CHAMPS-study. Separate multivariable Poisson regression models were used to assess the differences between groups for the outcome variables: contacts with physiotherapists, chiropractors, and general practitioners, and overall contacts.

**Results:**

Compared to National Controls, the CHAMPS-Group had fewer physiotherapy contacts before the study with an estimated mean of 0.01 vs 0.02 per person-year, and after (0.13 vs 0.18 per person-year), corresponding to a crude incidence rate ratio (IRR) of 0.69 (95% confidence intervals (CI): 0.58–0.83) after the study period. However, they had more chiropractor contacts before (0.05 vs 0.03), and after (0.21 vs 0.09) the study, with a crude IRR of 2.29 (95% CI: 1.93–2.71) after the study period. General practice contacts were equal for the CHAMPS-group compared to national controls (5.84 vs 5.84) before the study but reduced during and after (3.21 vs 3.71), with a crude IRR of 0.86 (95% CI: 0.83–0.90) after the study. Comparable patterns of contacts changes from before to after the study were observed between the CHAMPS-group and the Local Controls except for physiotherapy which was equal between the two groups after the study.

**Conclusion:**

Our findings suggest that research studies involving systematic engagement with participants experiencing musculoskeletal complaints can influence subsequent healthcare-seeking behaviour. Future research should address the influence of health literacy, health education, and healthcare provider recommendations on healthcare decisions during such research studies.

**Supplementary Information:**

The online version contains supplementary material available at 10.1186/s12875-023-02233-z.

## Background

Participating in research clinical studies often involves regular interactions with healthcare professionals, but the influence on the participant’s future help-seeking behaviour remains unclear.

Primary care providers, such as physiotherapists (PT), chiropractors (DC), and general practitioners (GPs), often treat pain and injuries. However, young people often choose to self-manage their pain and injuries without seeking professional help. Previous studies have shown that 51–60% of adolescents with pain seek help from healthcare professionals [1, 2].

Help-seeking and coping strategies may, to some extent, be learned behaviours [3, 4], as young people observe and internalize the ways in which adults around them cope with health problems and seek help [3, 4].

In the period 2008–2014, a longitudinal quasi-experimental trial among school students, “The Childhood Health Activity and Motor Performance School Study – Denmark” (CHAMPS) [5], was performed. The aim of the CHAMPS-study DK was to evaluate the effects of an increased number of (six versus two) physical education lessons on several health markers such as cardiovascular risk, cardiometabolic risks, physical fitness, pain and injuries among others [5].

Every week during the research period, parents responded if their child had experienced any musculoskeletal complaints during the preceding week. To secure proper treatment and supervision, a telephone consultation was conducted by a clinician within the research team with the parents of the children with musculoskeletal complaints. If deemed necessary, the child was offered an examination within the next 2 weeks [6]. In some cases, the child was referred for further paraclinical examination, such as X-ray, ultrasound, or MRI, and in some cases seen by a medical specialist [5, 7]. The sport school children had increased rate ratio of injuries the first 2.5 years: 1.29 (95% CI = 1.07–1.56 [8]. There were no differences in spinal injuries [9].

The participants may from their visits to these examinations somewhat have learned to deal with their musculoskeletal complaints and self-manage their pain or increased their dependency on healthcare professionals as a result of the study.

Therefore, we hypothesize that these study-specific systematic contacts with participants with musculoskeletal complaints provided by clinicians from the CHAMPS research team might influence the participants´ future healthcare-seeking behaviour.

In this study, we aimed to investigate if participating in the school-based study, CHAMPS, was associated with changed frequency of contacts with private physiotherapy (PT) and chiropractor (DC) clinics outside hospitals, and general practitioners (GP) in the following five-year-period compared to non-participating children as controls.

## Methods

### The CHAMPS-study DK

In 2007, the Svendborg Municipality Council in Denmark launched an initiative to improve the physical health of primary school students by increasing physical activities in public schools named “The Svendborg Project”. The initiative was scientifically evaluated by researchers from the University of Southern Denmark, leading to the Childhood Health Activity and Motor Performance School Study – Denmark (CHAMPS-study DK) which ran from 2008 to 2014 [5].

The CHAMPS-study Dk was a natural quasi-experimental study. School children were recruited in the municipality of Svendborg (58.600 inhabitants). All 19 primary schools in the municipality of Svendborg were invited to participate. 10 schools participated in this study, with six of these schools agreeing to participate as intervention schools, while four schools served as control schools. Schools were matched by size and socioeconomic distribution to ensure comparable catchment areas between the intervention schools and control schools.

The control schools maintained the mandatory two 45-minute physical education lessons per week (90 minutes in total). The sports schools incorporated an additional four physical education lessons, totalling 270 minutes weekly, for all children from pre-school (age 5) to the sixth grade (age 12). For those in the seventh to ninth grade (age 13–15), the number of additional physical education lessons was reduced to the mandatory two sessions per week [5]. Participation in the CHAMPS-study DK was voluntary for children attending the included schools. Additional details regarding the study sample and procedures have been reported previously [5, 10, 11].

### Study design and population

We employed a register-based cohort design, wherein we tracked the healthcare utilisation of three groups of children over a period of 17–21 years. We compared register-based outcomes between the CHAMPS participants born in 1998–2002 (CHAMPS-group) and both a primary and a secondary control group identified by the registry of Statistic Denmark. All CHAMPS participants were included regardless of their school intervention status or their own compliance.

As participation in the CHAMPS-study was self-selected at both the school and individual levels, we included both a national (primary) and local (secondary) control group to gain a more comprehensive understanding of the factors that may influence the result of our study.

**Fig. 1:**
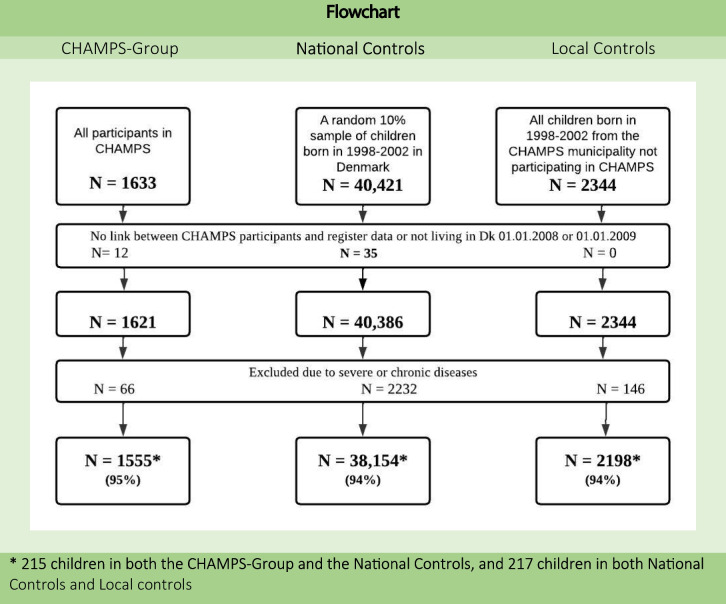
Flowchart of study population

The primary control group, National Controls, consisted of a random 10% sample of Danish children of the same age as the CHAMPS-study children born in 1998–2002 in Denmark (eligible for inclusion in the CHAMPS-Group). The secondary control group, Local Controls, comprised children from the municipality of Svendborg born in 1998–2002 who did not participate in the CHAMPS-study (Fig. 1).

All Danish citizens and all permanent residents of Denmark are assigned a unique personal identification number (CPR.NR) [12], which the included participants had to possess. Furthermore, they should have resided in Denmark on 1 January 2008 and/or 1 January 2009. Children from all three groups with a chronic or severe disease that could result in recurrent visits to the hospital or other healthcare professionals were identified with a relevant diagnostic code in The Danish National Patient Register between birth and 31.10.2008 and were excluded (List S1).

In Denmark, public medical assistance is provided free of charge for all Danish residents, and more than 98% of the Danish population is registered with a specific general practice [13].

General Practice play a central role in the healthcare system and acts as the primary entry point for healthcare services, overseeing both disease management and preventive measures. Each resident in Denmark has the privilege of consulting a GP at no cost for any healthcare concern [14]. GPs have the authority to make referrals to secondary healthcare services, including diagnostic imaging and specialised care, as well as to specialised primary healthcare providers like physiotherapists.

At private clinics outside hospitals, patients can directly access physiotherapy services, which incurs a full-service fee. This fee can be paid by the patient or covered by private health insurance. However, when referred by a GP, patients are eligible for partial reimbursement, typically around 40%, for physiotherapy services provided by licensed providers in Denmark’s regions. The remaining cost is the responsibility of the patient or can be covered by private health insurance. While physiotherapists do not possess official referral rights, they can suggest referrals (e.g., to advanced imaging) through electronic communication with GPs.

Chiropractic services can also be directly assessed by patients, with approximately 20% of the cost reimbursed, regardless of a GP referral. Patients are responsible for the remaining cost or can use private health insurance for coverage. Chiropractors have the authority to refer patients for advanced imaging, and the majority have in-house radiography equipment.

**Fig. 2:**
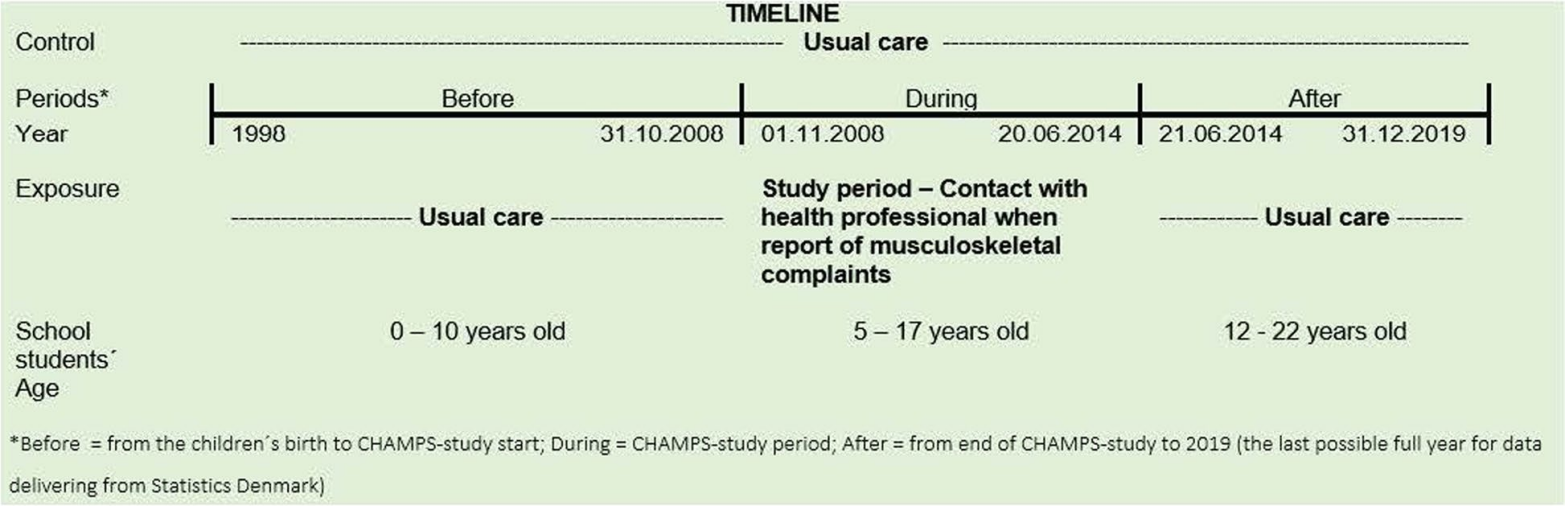
Study timeline

We included register-based outcomes in three different periods defined based on CHAMPS: before CHAMPS (1998–31.10.2008), during (01.11.2008–20.06.2014), and after (21.06.2014–31.12.2019). The year 1998 was chosen as this was the birth year of the oldest included year-group of children (Fig. 2).

### Data sources

We utilized longitudinal data spanning from 1998 to 2019, covering contacts with the Danish healthcare system.

For all the children included in this study and their parents, register data were obtained through Statistics Denmark. Statistics Denmark is a governmental institution responsible for collecting electronic records for a various statistical and scientific purposes. The unique CPR.NR [12] is employed in all health records, allowing for seamless linkage between different registries.

For this study, Statistics Denmark provided data from two key sources: the Danish National Health Service Register [15] and the Danish National Patient Register [12]. Additionally, we incorporated data from various registers related to economic factors, education, housing conditions, internal migrations within Denmark, as well as immigration and emigration. Finally, we connected the participants’ personal identification number from the CHAMPS-study DK to the register data.

 The Danish National Health Service Register [15] contains high-quality data gathered for administrative and scientific purposes from health service contractors in primary healthcare. The register encompasses information regarding citizens, healthcare providers, and health services, although it contains minimal clinical information. We specifically utilized data related to contacts with PTs, DCs, and GPs. It is important to note that Data from healthcare providers who do not have any payment contract with the regions in Denmark are not registered in the Danish national registers.

 The Danish National Patient Register [12] include administrative data on all somatic hospital admissions since 1977, and since 1995, it has also covered contacts with outpatient clinics and psychiatric inpatients services. Starting from 2007, it encompasses data on all inpatient and outpatient contacts, including diagnostic codes, examinations, and treatments for all somatic, psychiatry, and private hospital admissions, as well as interactions with emergency departments.

We specifically used data from diagnostic codes to identify and exclude children who had chronic and severe illness during the period before the CHAMPS-study DK, as such conditions could lead to recurring visits to hospitals or other healthcare professionals.

### Analysis

For the descriptive part of this study, primary public healthcare usage was reported as the mean number of contacts with primary care per person-year. Healthcare usage was reported for each group and within different time-periods, divided into PT, DC, and GP, as well as the total contacts. (Details on specific codes are included in supplementary (List S2)).

To assess the differences in healthcare usage between the CHAMPS-Group and the two control groups for the outcome variables (contacts with PTs, DCs, and GPs, and overall contacts), separate multivariable Poisson regression models of incidence rate (IR) were employed. Both crude and adjusted analyses were conducted. Note that the crude model estimates lead to the mean rates described above. The primary exposure variable was defined as group status, specifically, participation in CHAMPS or non-participation in CHAMPS. The risk time was determined based on the three study periods. Participants entered the study at birth and were censored at the study end, death, or emigration.

In the adjusted models, we included gender, parental healthcare usage before CHAMPS, and equivalised disposable family income. Regarding parental healthcare usage, due to varying numbers of parents registered for each child (ranging from 1 to 4), we included data from one parent for each child with whom the child lived. Parental healthcare usage was defined as follows: for each outcome during the period before CHAMPS, we included the number of contacts with GPs, DC, and PT (outside hospitals) of the child’s parent or guardian who had the highest utilization of these services and lived with the child. Subsequently, we grouped contacts with GPs and total contacts into tertiles of low, middle, and high usage, while contacts with PTs and DCs were categorised into two groups (low, defined as 0 contacts, and high, defined as > 0 contacts). Equivalised disposable income per period was also grouped into tertiles.

## Results

Data from 41,907 children and their parents were available for this study (Fig. 1).

There were no differences observed in gender distribution among the three groups (Table 1).

**Table 1:** Gender, baseline parents´ healthcare usage, and equivalised disposable income (adjustment variables) before, under and after CHAMPS for the CHAMPS-Group, National Controls and Local Controls

Variable		CHAMPS-Group N (%)	National Controls N (%)	Local Controls N (%)
Period 1: Before (1998–2008) *				
**Overall N**		1550	38,154	2198
**Gender**	Girls	808 (52.1%)	18,717 (49.1)	1090 (49.6)
	Boys	742 (47.9)	19,437 (50.9)	1108 (50.4)
**Income**^**a**^ **(tertiles)**	Low	393 (25.4)	12,650 (33.2)	925 (42.1)
	Middle	656 (42.3)	12,527 (32.8)	784 (35.7)
	High	501 (32.3)	12,977 (34.0)	489 (22.2)
**Parents’ healthcare usage**^**b**^ **(tertiles)**				
**Physiotherapy**^**c**^	Low	1036 (69.0)	25,024 (66.5)	1478 (67.9)
	High	466 (31.0)	12,633 (33.5)	698 (32.1)
**Chiropractic**^**c**^	Low	972 (64.7)	27,880 (74.0)	1468 (67.5)
	High	530 (35.3)	9777 (26.0)	708 (32.5)
**General practice**	Low	454 (30.2)	12,735 (33.8)	590 (27.1)
	Middle	464 (30.9)	12,645 (33.6)	669 (30.7)
	High	584 (38.9)	12,277 (32.6)	917 (42.1)
**Total**	Low	405 (27.0)	12,802 (34.0)	572 (26.3)
	Middle	443 (29.5)	12,692 (33.7)	643 (29.5)
	High	654 (43.5)	12,163 (32.3)	961 (44.2)
Period 2: During (2009–2014) *				
**Overall N**		1555	37.938	2182
**Gender**	Girls	808 (52.0)	18,603 (49.0)	1083 (49.6)
	Boys	747 (48.0)	19,335 (51.0)	1099 (50.4)
**Income**^**b**^ **(tertiles)**	Low	424 (27.3)	12,595 (33.2)	873 (40.0)
	Middle	593 (38.1)	12,526 (33.0)	773 (35.4)
	High	538 (34.6)	12,817 (33.8)	536 (24.6)
Period 3: After (2014–2019) *				
**Overall N**		1553	37,730	2176
**Gender**	Girls	807 (52.0)	18,499 (49.0)	1082 (49.7)
	Boys	746 (48.0)	19,231 (51.0)	1094 (50.3)
**Income**^**b**^ **(tertiles)**	Low	396 (25.5)	12,506 (33.1)	918 (42.2)
	Middle	586 (37.7)	12,483 (33.1)	751 (34.5)
	High	571 (36.8)	12,741 (33.8)	507 (23.3)

^a^Tertiles were used to divide the equivalised disposable income in low, middle, and high

^b^Contacts per person-year. Tertiles were used to divide parents´ healthcare usage per person per year in low, middle, and high based on the children’s parent or guardian with the highest use of healthcare services who the child lived with

^c^Due to the proportion of parents with none contacts with physiotherapy and chiropractic estimated tertiles results in only lower and upper

* Cut date for 2008: 31.10.2008/01.11.2008, and for 2014: 20.06.2014/21.06.2014

The proportion of participants from low-income families was lower in the CHAMPS-Group than in the two control groups, while the proportion of middle-income families was higher. The proportion of participant with high-incomes was similar between the CHAMPS-Group and National Controls. Additionally, the proportion of parents with high-healthcare-usage was greater in the CHAMPS-Group compared to the National Controls but slightly lower compared to Local Controls during the period before CHAMPS (Table 1).

**Fig. 3:**
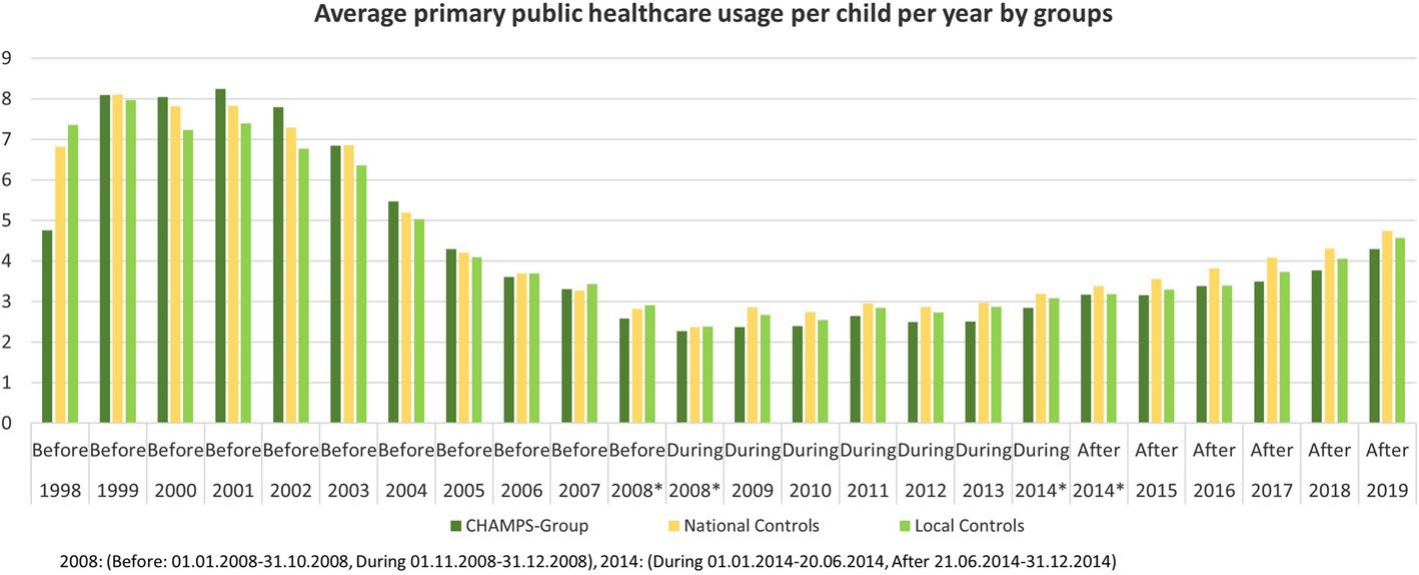
Mean primary public healthcare usage per child per year by groups

In all three groups, the majority of the healthcare services were recorded as contacts with GPs e.g., among the 38,000 National Controls in the five-year period after CHAMPS, there were 774,584 contacts with GPs, in contrast to 37,919 contacts with PTs and 19,533 contacts with DCs (Table 2). The distribution of healthcare usage in each study-year is illustrated in Fig. 3.

**Table 2:** Mean* contacts (per person year) to primary public health care before, under and after CHAMPS

	CHAMPS-Group	National Controls	Local Controls
Period 1: Before (1998–2008) **			
**Overall N**	1550	38,154	2198
**Risk time (Person years)**	12,848	329,485	19,624
**Contacts with physiotherapists (n)**	123	7173	689
**Contacts with chiropractors (n)**	673	9473	854
**Contacts with general practitioners (n)**	75,070	1,923,236	111,319
**Total contacts (n)**	75,866	1,939,882	112,862
Contacts with physiotherapists, Mean*	0.01	0.02	0.04
Contacts with chiropractors, Mean*	0.05	0.03	0.04
Contacts with general practitioners, Mean*	5.84	5.84	5.67
Overall contacts, Mean*	5.91	5.89	5.75
Period 2: During (2008–2014)**			
**Overall N**	1555	37,938	2182
**Risk time (Person years)**	8766	213,710	12,300
**Contacts with physiotherapists (n)**	498	22,921	1578
**Contacts with chiropractors (n)**	1240	10,979	1212
**Contacts with general practitioners (n)**	20,311	584,268	31,069
**Total contacts (n)**	22,049	618,012	33,851
Contacts with physiotherapists, Mean*	0.06	0.11	0.13
Contacts with chiropractors, Mean*	0.14	0.05	0.10
Contacts with general practitioners, Mean*	2.32	2.73	2.53
Overall contacts, Mean*	2.52	2.89	2.75
**Period**: After (2014–2019)**			
**Overall N**	1553	37,730	2176
**Risk time (Person years)**	8590	208,639	12,033
**Contacts with physiotherapists (n)**	1078	37,919	1550
**Contacts with chiropractors (n)**	1838	19,533	1903
**Contacts with general practitioners (n)**	27,560	774,584	41,226
**Total contacts (n)**	30,476	831,917	44,679
Contacts with physiotherapists, Mean*	0.13	0.18	0.13
Contacts with chiropractors, Mean*	0.21	0.09	0.16
Contacts with general practitioners, Mean*	3.21	3.71	3.43
Overall contacts, Mean*	3.55	3.99	3.71

* Sum of all contacts in period/sum of risk time in period (unit: years)/ per person

** Cut date for 2008: 31.10.2008/01.11.2008, and for 2014: 20.06.2014/21.06.2014

In all three periods, the children in the CHAMPS-Group had fewer contacts with PTs than the National Controls with an estimated mean of 0.01 contacts per person-year before vs 0.02, and of 0.13 vs 0.18 after, corresponding to a crude incidence rate ratio (IRR) of 0.69 (95% confidence intervals (CI)**:** 0.58–0.83) in the after period. For CDs, the mean number contacts per person-year were consistently higher for the CHAMPS-group than National Controls in all periods, with 0.05 vs 0.03 before, and 0.21 vs 0.09 after, corresponding to a crude IRR of 2.29 (95% CI: 1.93–2.71) in the after period. The estimated mean number of contacts per person-year with GPs was equal between the National Controls and the CHAMPS-group before the CHAMPS-study period but changed to fewer during and after with 3.21 vs 3.71 after, corresponding to a crude IRR of 0.86 (95% CI: 0.83–0.90).

In total, the CHAMPS-Group had slightly more contacts than the National Controls in the before period with an estimated mean of 5.91 contacts per person-year vs 5.89 and fewer during and after with an estimated mean of 3.55 vs 3.99 after, corresponding to a crude IRR of 0.89 (95% CI: 0.86–0.92) (Tables 2 and 3).

**Table 3:** Associations between group-status and primary healthcare usage. Separate multivariable Poisson models are fitted for the four outcomes: overall number of contacts, contacts with GP, physiotherapist, or chiropractor. Presented are estimated incidence rate ratios (IRR) with corresponding 95% confidence intervals (CI) for the children in the CHAMPS-Group against reference National Controls. Adjustment factors were Children’s gender, parents healthcare^a^ use in the period before CHAMPS-study DK, and the equivalised disposable family income^b^

	National Controls											
	Contacts with physiotherapist			Contacts with chiropractor			Contacts with General Practice			Overall contacts		
	IRR	95% CI	*p*-value	IRR	95% CI	*p*-value	IRR	95% CI	*p*-value	IRR	95% C)	*p*-value
**Period 1: Before (1997–2008)**												
**Crude model**												
**Constant (National controls)**	0.02	0.02–0.03	< .001	0.03	0.03–0.03	< .001	5.84	(5.80–5.87)	< .001	5.89	5.85–5.92	< .001
**Group (ref national controls)**	0.44	0.25–0.77	.004	1.82	1.50–2.21	< .001	1.00	0.97–1.03	.94	1.00	0.98–1.03	.83
**Adjusted model**												
**Group (ref national controls)**	0.44	0.25–0.78	<.005	1.54	1.27–1.86	< .001	0.99	0.96–1.01	.27	0.95	0.93–0.98	< .001
**Gender (ref boys)**	0.80	0.50–1.26	.33	0.84	0.76–0.93	< .001	0.94	0.93–0.95	< .001	0.95	0.94–0.96	< .001
**Income (ref low)**												
**Middle**	1.09	0.64–1.87	.75	1.59	1.37–1.84	< .001	1.06	1.05–1.08	< .001	1.09	1.08–1.10	< .001
**High**	1.58	0.95–2.63	.08	1.82	1.57–2.10	< .001	1.05	1.03–1.06	< .001	1.10	1.09–1.11	< .001
**Parents´ healthcare (ref low)**												
**Middle**	–	–	–	–	–	–	1.12	1.10–1.13	< .001	1.37	1.36–1.39	< .001
**High**	1.46	0.93–2.28	.10	3.92	3.54–4.34	< .001	1.42	1.40–1.44	< .001	1.82	1.80–1.85	< .001
**Period 2: During (2009–2014)**												
**Crude model**												
**Constant (National controls)**	0.11	0.10–0.12	< .001	0.05	0.05–0.05	< .001	2.73	2.71–2.75	< .001	2.89	2.87–2.92	< .001
**Group (ref national controls)**	0.53	0.39–0.72	< .001	2.75	2.30–3.30	< .001	0.85	0.82–0.88	< .001	0.87	0.84–0.90	< .001
**Adjusted model**												
**Group (ref national controls)**	0.53	0.39–0.72	< .001	2.66	2.22–3.18	< .001	0.85	0.82–0.88	< .001	0.87	0.84–0.90	< .001
**Gender (ref boys)**	1.07	0.86–1.34	.52	1.19	1.07–1.34	<.002	1.15	1.13–1.17	< .001	1.15	1.13–1.17	< .001
**Income (ref low)**												
**Middle**	1.03	0.75–1.42	.86	1.88	1.60–2.21	< .001	0.94	0.92–0.96	< .001	0.96	0.93–0.98	< .001
**High**	1.36	1.01–1.84	.04	2.30	1.98–2.67	< .001	0.91	0.90–0.93	< .001	0.94	0.92–0.96	< .001
**Period 3: After (2014–2019)**												
**Crude model**												
**Constant (National controls)**	0.18	0.17–0.19	< .001	0.09	(.09–0.10	< .001	3.71	3.68–3.74	< .001	3.99	3.95–4.02	< .001
**Group (ref national controls)**	0.69	0.58–0.83	< .001	2.29	1.93–2.71	< .001	0.86	0.83–0.90	< .001	0.89	0.86–0.92	< .001
**Adjusted model**												
**Group (ref national controls)**	0.67	0.56–0.80	< .001	2.17	1.83–2.57	< .001	0.86	0.83–0.89	< .001	0.88	0.85–0.91	< .001
**Gender (ref boys)**	1.65	1.45–1.89	< .001	1.47	1.35–1.60	< .001	1.84	1.82–1.87	< .001	1.82	1.79–1.85	< .001
**Income (ref low)**												
**Middle**	1.15	0.96–1.38	.12	1.75	1.56–1.97	< .001	0.90	0.88–0.92	< .001	0.92	0.90–0.94	< .001
**High**	1.29	1.09–1.52	< .01	2.12	1.90–2.38	< .001	0.87	0.85–0.89	< .001	0.90	0.88–0.92	< .001

^a^Tertiles were used to divide parents´ healthcare usage in low, middle, and high based on the children’s parent or guardian with the highest use of healthcare services who the child lived with

^b^Tertiles were used to divide the equivalised disposable income in low, middle, and high. Before: Low: < 141.742 d.kr, Middle: 141.742–181.649 d.kr., High: > 181.649 d.kr., During: Low: < 184.573 d.kr, Middle: 184.573–251.479 d.kr., High: > 249.471 d.kr., After: Low: < 193,409 d.kr, Middle: 193409–279,279 d.kr., High: > 279,279 d.kr

**Fig. 4:**
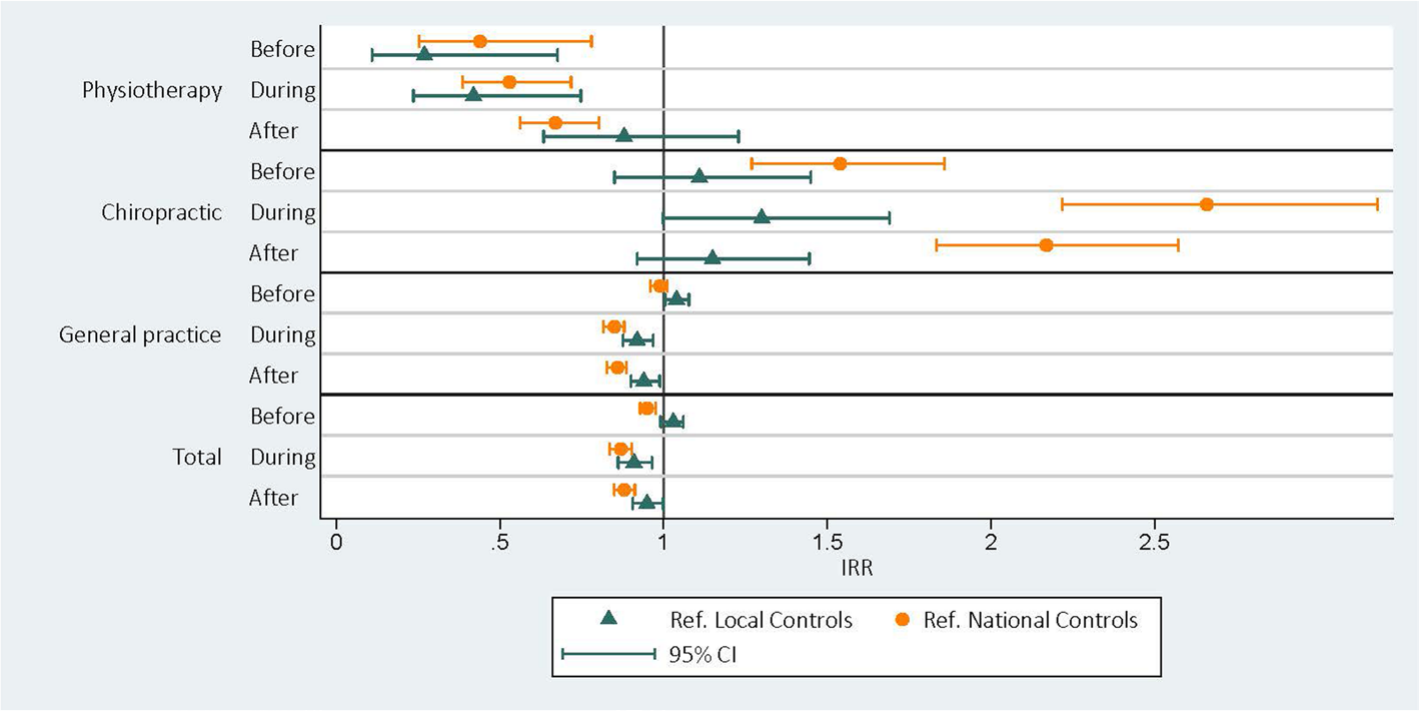
Estimated incidence rate ratios (IRR) with corresponding 95% confidence intervals (CI) between the CHAMPS-group and the two control groups (ref). Separate multivariable Poisson models are fitted for the four outcomes: contacts with physiotherapist, chiropractor, or General Practitioners and total number of contacts. Adjustment factors were children’s gender, parent’s healthcare^a^ usage in the period before CHAMPS, and the equivalised disposable family income^b^

In the adjusted models comparing the CHAMPS-group to the National Controls (Fig. 4, Table 3), the incidence rate ratios (IRR (95% CI)) increased from the before period to during and after for both PT and DC, with statistically significant results in all three periods. PT: before 0.44 (0.25–0.78), during 0.53 (0.39–0.72) and after 0.67 (0.56–0.80), and DC: before 1.54 (1.27–1.86), during 2.66 (2.22–3.18), and after 2.17 (1.83–2.57). For GP and Total contacts, the adjusted IRR (95% CI) decreased from the before period to during and after, with statistically significant results for all three periods (except for GP before). GP: before 0.99 (0.96–1.01), during 0.85 (0.82–0.88), after 0.86 (0.83–0.89), Total contacts: before 0.95 (0.93–0.98), during 0.87 (0.84–0.90), after 0.88 (0.85–0.91) (Fig. 4, Table 3).

Similar associations for PT and GP contacts were found between the CHAMPS-group and the Local Controls (Fig. 4, Table 4), with statistically significant differences for PT before and during CHAMPS and for GP in all periods. PT: before 0.27 (0.11–0.68), during 0.42 (0.24–0.75) and after 0.88 (0.63–1.23), and GP: before 1.04 (1.01–1.08), during 0.92 (0.88–0.97), and after 0.94 (0.90–0.99).

**Table 4:** Associations between group-status and primary healthcare usage. Separate multivariable Poisson models are fitted for the four outcomes: overall number of contacts, contacts to GP, physiotherapist, or chiropractor. Presented are estimated incidence rate ratios (IRR) with corresponding 95% confidence intervals (CI) for the children in the CHAMPS-Group against reference Local Controls. Adjustment factors were Children’s gender, parents healthcare^a^ use in the period before CHAMPS-study DK, and the equivalised disposable family income^b^

	Local Controls											
	Contacts to physiotherapist			Contacts to chiropractor			Contacts to General Practice			Overall contacts		
	IRR	95% CI	*p*-value	IRR	95% CI	*p*-value	IRR	95% CI	*p*-value	IRR	95% C)	*p*-value
**Period 1: Before (1997–2008)**												
**Crude model**												
**Constant (Local controls)**	0.04	(0.02–0.07)	< .001	0.04	0.04–0.05	< .001	5.67	5.54–5.81	< .001	5.75	5.61–5.89	< .001
**Group (ref local controls)**	0.27	(0.11–0.66)	.04	1.20	0.93–1.56	.16	1.03	0.99–1.07	.11	1.03	0.99–1.06	.15
**Adjusted model**												
**Group (ref local controls)**	0.27	0.11–0.68	< .01	1.11	0.41–1.11	.44	1.04	1.01–1.08	.02	1.03	0.99–1.06	.14
**Gender (ref boys)**	1.00	0.31–3.27	1.00	0.81	0.63–1.05	.11	0.96	0.93–0.99	.01	0.96	0.93–1.00	.03
**Income (ref low)**												
**Middle**	0.49	0.15–1.64	.25	1.28	0.91–1.80	.15	1.04	1.00–1.08	.07	1.05	1.00–1.09	.03
**High**	1.43	0.32–6.41	.64	1.36	0.95–1.96	.09	1.02	0.98–1.07	.29	1.05	1.00–1.10	.03
**Parents´ healthcare (ref low)**												
**Middle**	–	–	–	–	–	–	1.12	1.07–1.18	< .001	1.30	1.24–1.36	< .001
**High**	1.91	0.57–6.45	.30	3.59	2.75–4.68	< .001	1.39	1.33–1.45	< .001	1.67	1.60–1.74	< .001
**Period 2: During (2009–2014)**												
**Crude model**												
**Constant (Local controls)**	0.13	0.08–0.22	< .001	0.10	0.08–0.12	< .001	2.53	2.44–2.61	< .001	2.75	2.64–2.87	< .001
**Group (ref local controls)**	0.44	0.24–0.81	< .01	1.44	1.11–1.86	< .01	0.92	0.87–0.96	< .001	0.91	0.86–0.97	< .002
**Adjusted model**												
**Group (ref local controls)**	0.42	0.24–0.75	< .01	1.30	1.00–1.69	.05	0.92	0.88–0.97	< .01	0.91	0.86–0.97	< .002
**Gender (ref boys)**	2.22	1.12–4.44	.02	1.12	0.87–1.46	.38	1.18	1.13–1.24	< .001	1.21	(1.14–1.28)	< .001
**Income (ref low)**												
**Middle**	0.83	0.27–2.55	.74	2.24	1.53–3.29	< .001	0.95	0.90–1.01	.10	0.98	0.91–1.05	.52
**High**	1.44	0.56–3.71	.45	2.54	1.76–3.67	< .001	0.93	0.87–0.99	.03	0.98	0.91–1.06	.68
**Period 3: After (2014–2019)**												
**Crude model**												
**Constant (Local controls)**	0.13	0.10–0.17	< .001	0.16	0.14–0.18	< .001	3.43	3.31–3.54	< .001	3.71	3.58–3.85	< .001
**Group (ref local controls)**	0.97	0.71–1.34	.87	1.35	1.08–1.69	.08	0.94	0.89–0.98	.01	0.96	0.91–1.01	.08
**Adjusted model**												
**Group (ref local controls)**	0.88	0.63–1.23	.46	1.15	0.92–1.45	.22	0.94	0.90–0.99	<.02	0.95	0.91–1.00	<.05
**Gender (ref boys)**	1.93	1.38–2.69	< .001	1.08	0.86–1.34	.51	1.85	1.77–1.94	< .001	1.80	1.72–1.89	< .001
**Income (ref low)**												
**Middle**	1.32	0.87–2.00	.19	2.01	1.50–2.69	< .001	0.92	0.87–0.97	< .01	0.96	0.90–1.01	.14
**High**	1.78	1.07–2.98	.03	3.10	2.32–4.14	< .001	0.87	0.82–0.92	< .001	0.95	0.89–1.01	.09

For DC the results were different. The adjusted IRR (95% CI) between the CHAMPS-Group and the Local Controls increased from 1.11 (0.41–1.11) (before) to 1.30 (1.00–1.69) (during) and 1.15 (0.92–1.45) (after), with statistically nonsignificant results for all periods (Fig. 4, Table 4).

## Discussion

We aimed to investigate the influence of participation in the CHAMPS-study on children’s healthcare seeking behaviour in comparison to non-participating controls. The main finding of our research was a reduction in healthcare seeking and a shift in preferences among the children who participated in CHAMPS compared to both control groups, and these changes persisted for at least 5 years after the study had ended.

In comparison to National Controls, children in the CHAMPS-group had 56% fewer contacts per person-year (adjusted IRR) with PTs before the study, and 33% fewer contacts after the study. Conversely, their contacts with DCs increased from 54% more contacts before the study to 117% more contacts after study end compared to National Controls, and the number of GP contacts changed from 1% fewer before CHAMPS to 14% fewer after for CHAMPS children as compared to National controls. This pattern was reflected in the overall number of total contacts, with children in the CHAMPS-group having 12% fewer contacts corresponding to half a visit less per child per year after the study.

A similar association was observed when comparing the CHAMPS-group to Local Controls, although the differences were less pronounced. These findings suggest that participating in CHAMPS was associated with changes in contacts with the primary public healthcare services among participants. Some CHAMPS participants altered their healthcare-seeking preferences, opting for PTs and DCs over GPs after the end of the CHAMPS-study.

While a decrease of half a GP visit per year and an increase of 0.1 DC visit per year may not have significant clinical implications for individual children, the cumulative effect could have a considerable impact on both healthcare providers and the health-care system as a whole.

In our study, these changes can be seen as both positive and negative outcomes. In the Danish healthcare system, the roles of different healthcare professions vary significantly with GPs often serving as gatekeepers and diagnosticians, traditionally referring patients with musculoskeletal complaints to PTs and, to a lesser extent, DCs for treatment [16]. It is plausible that CHAMPS participants and their parents gained a level of health education during the study, enabling them to self-manage their complaints and adequately address their healthcare needs. It is generally accepted that many musculoskeletal complaints and injuries naturally resolve without the need for any healthcare intervention [17], highlighting the potential of overuse of healthcare services. This could explain why some CHAMPS-children experiencing musculoskeletal complaints post-study did not seek their GPs, PTs or DCs, while others may have bypassed GP visits and sought care from PTs and DCs. However, in some cases the lack of visits to the general practitioner could be concerning, as it may result in overlooked serious illnesses with musculoskeletal symptoms. In these situations, not consulting the general practitioner would constitute a negative outcome of participating in the CHAMPS-study and could present a significant ethical dilemma.

To our knowledge, the potential influence of participating in a research study on patients’ future healthcare seeking behaviour has not been previously investigated. This concept, however, bears some resemblance to the well-described seeding trails´ [18], which are clinical studies designed by pharmaceutical companies primarily intending to influence the participating physicians´ prescribing behaviour to promote the use of drugs that were recently approved or are under review by regulatory authorities [18, 19]. In the CHAMPS-study DK, the decision to offer healthcare services as part of the study design was made with the intention of securing proper treatment and ensuring adherence and engagement in the study [5]. While we did not have information on the healthcare providers´ recommendations regarding patients´ healthcare-seeking behaviour, we assume that variations in recommendations by the research team could have impacted the choices made by participants. In the CHAMPS-study DK, the research team responsible for following up with children who reported musculoskeletal complaints consisted of a varying number of physiotherapists and chiropractors and one medical practitioner [5]. The disproportionate number of physiotherapists and chiropractors compared to general practitioner trainees on the research team may have unintentionally influenced the children (and their parents) to seek PT and DC subsequently. The increased utilization of PT and DC services among CHAMPS children could, therefore, be seen as a qualified preference for more direct asses to right healthcare but may also reflect an unintended influence by the clinicians in the research team.

### Strength and limitations

The main strength of this study lies in its relatively large sample size and comprehensive nationwide approach, utilising register data that covers the study groups over a period of 22 years. Our data material is robust, encompassing high-quality information on all investigated outcomes both before, during, and after a five-year experimental study, along with key adjustment variables such as income, gender, and parental healthcare utilisation.

Our primary outcomes were the numbers of contacts with PTs, DCs, and GPs. As registering contacts is mandatory in Denmark and essential for payment, the overall completeness of these data are high [15]. However, visit to private PT clinics without payment contracts with the regional health authorities in Denmark are not registered in the Danish national databases, unlike visits to the public clinics. Private PT clinics are common in Denmark, but specific data on this parameter is not available. A recent report from 2020 made by VIVE (The Danish Center for Social Science Research) [20] estimate the number of such clinics in Denmark to be between 130 and 740 compared to 576 contract clinics with varying numbers of PTs engage in each clinic. Therefore, the number of PT visits may be underestimated in our study. The report also estimated that there is a higher proportion of PTs involved in PT clinics per citizen in the Region of Southern Denmark compared to the Region Sjælland, but we do not have specific data on this parameter either. As the national controls are a random sample of all regions in Denmark, the average number of PTs per citizen in this group might be a bit lower than for the Municipality of Svendborg, but it is reasonable to assume that this potential small difference is consistent through all three periods and therefore cannot significantly impact our main result.

Additionally, our study did not directly measure participants´ access to healthcare services, including the availability of PT, DC, and GP services in their geographic area. Variations in access to these services may have influenced participants´ choices, which we were unable to account for in our analysis.

The primary limitation of this study stems from its observational design. Despite having access to comprehensive healthcare utilisation data for children before, during, and after the CHAMPS-study, and employing two control groups, we acknowledge that we probably cannot establish causal relationships. Instead, we can only identify associations followed by hypothetical explanations. However, the study could also be regarded as a real-world or what might be termed a “phase four study”. In such studies, the practical implications of an intervention are assessed in real-world conditions. Randomized studies often exhibit weakness due to their homogenous study populations, causing the effectiveness of a given intervention to diminish in a real-world setting, which tends to be more heterogenous than the typical study population” in a randomized controlled trial.

Identifying the optimal control group posed challenges. The CHAMPS-study involved self-selection from the schools, and we lacked data explaining why 9 schools declined to participate. Consequently, we cannot rule out that non-participating children from the municipality of Svendborg might be systematically different from those included in the study. Ideally, an optimal control group should have no awareness of the trial. Hence, we opted to incorporate a national control group consisting of a random 10% sample of Danish children to mitigate this potential bias. However, while all Danish citizens have equal right to healthcare access, it is important to acknowledge that the difference in healthcare service access between Svendborg and the national controls may not be fully accounted for in our analysis. This discrepancy could result in a “Svendborg effect” rather than an effect solely attributed to participation in the CHAMPS-study. Therefore, as an optimal control group was challenged to identify, we chose to include both. Nevertheless, it is worth noting that the most significant change observed was a decrease in GP services, which alone cannot attribute to a shortage of GP clinics, as there are fewer clinics in rural Denmark than in Svendborg. We could have considered including comparable municipalities instead of a random 10% sample as controls, but this would limit the generalizability of our study. In summary, both control groups present advantages and disadvantages, and variations in healthcare service admission may indeed vary locally. However, if Svendborg exhibits a systematic bias in either over- or underutilization of healthcare services, this bias should remain consistent across all three periods, which is not the case.

In the Local control group, there was a risk of biased participants because some of the children attended the schools included in CHAMPS and were, therefore, exposed the CHAMPS culture at school. This may explain the smaller difference in healthcare seeking between the CHAMPS-Group and the Local Controls compared to the difference between CHAMPS and the National Controls. Due to the exclusion of children with severe or chronic disease in CHAMPS we had to identify comparable children in both control groups. As we used specific diagnosis to exclude participants, there was a risk of information bias due to variation in coding, but this risk is likely to be minimal, as we only used the diagnosis codes for severe and chronic diseases.

The children in CHAMPS had varying numbers of complaints, and consequently, they were exposed to different rates of clinician contacts during the study period. This may lead to an underestimation of the subsequent change in healthcare seeking behaviour, as many of the included children did not report any or only a few musculoskeletal complaints during the five-year CHAMPS period.

We observed a skewed proportion of economic status between the children in the CHAMPS-Group and the control groups. Combined with the fact that payment for DC and PT services in Denmark combines user payment and public subsidies, this could contribute to difference in the usage of PT and DC services among the groups. However, we adjusted for economic status in our analyses, as we had register data available.

We acknowledge that our study lacked comprehensive information on the controls´ health and lifestyle factors, such as diet, physical activity, and smoking habits. These unmeasured variables could have influenced both participants healthcare-seeking behaviour and their musculoskeletal complaints, hence potentially confounding our results. Furthermore, we did not have data on participants´ quality of life, differences in pain levels, or the extent of patient education they may have received during the CHAMPS-study. This lack of information limits our ability to evaluate whether the children in CHAMPS achieved better health, changed their lifestyle, or changed their preference for seeking the three types of healthcare professionals. If they have achieved better health, this could explain the decrease in contact with the general practitioner after study end. Furthermore, if the CHAMPS-children adopted a more physical active lifestyle, this might potentially lead to more visits to the chiropractors or physiotherapists, due to increased risk of musculoskeletal complaints and injuries. However, it is also possible that they developed a higher ability to navigate the healthcare system.

Furthermore, it is essential to acknowledge that our study lack specific data on individual diagnosis and treatment details, making it challenging to directly assess the extent of overdiagnosis and overtreatment. Furthermore, we did not investigate the potential for overtreatment during the CHAMPS-study, where individuals may have received more medical attention than necessary due to their participation in the research study and the fact that they were systematically contacted if they reported any new musculoskeletal complaint.

### Consideration of generalisability

The findings of this study suggest that the impact of participating in a large-scale, municipality-based intervention, similar to the CHAMPS-study, may extend beyond our specific context. It is plausible that other municipalities can achieve comparable changes in health behaviour given the commonalities in the healthcare infrastructure, school settings, and sociodemographic factors that influence children’s lifestyles.

Furthermore, the observed changes are not necessarily limited to children with musculoskeletal complaints within the Danish healthcare system. The broader implication is that the effects of interactions with healthcare professionals may extend to all participants in studies that involve assessments and interactions with healthcare professionals according to national healthcare guidelines. It is important that researchers be aware of participating in their study might change the participants´ behaviour way beyond the study period.

## Conclusions

In conclusion, our findings suggest that children who participated in the CHAMPS-study showed a change in their healthcare-seeking preferences. The utilisation of physiotherapy and chiropractic services increased compared to the National Controls during the post-CHAMPS period while the utilisation of general practitioner services declined, simultaneously. This shift in healthcare-seeking behaviour persisted for at least 5 years after the collection of musculoskeletal complaints data and may be attributed to for example improved health, increased ability to make informed health choices, or the influence of study clinicians.

Our findings suggest that research studies involving systematic engagement of healthcare professionals with participants experiencing musculoskeletal complaints may have an impact on the subsequent healthcare-seeking behaviour of those participants. To explore this further, future studies could be designed to study the influence of health literacy, health education, and healthcare provider recommendations on the participant’s healthcare decisions. Often, the unintended influence on participants subsequent health care behaviour is not explicitly addressed during participant inclusion. Our study imply that future studies should include these issues when pursuing the ethical considerations.

### Supplementary Information

**Additional file 1.**

## Data Availability

We are according to the EU and Danish data protection legislation not allowed to submit the data or give access to the data used for the analyses. However, researchers who meet the criteria for access to confidential data, might apply for access directly to the Danish National Health and Medicines Authority in Denmark, e.g. via https://sundhedsdatastyrelsen.dk/da/forskerservice or https://www.dst.dk/da/TilSalg/Forskningsservice.

## Abbreviations

CHAMPS-study DK: “The Childhood Health Activity and Motor Performance School Study – Denmark”
CPR.NR: A unique personal identification number
PT: Physiotherapist
DC: Chiropractor
GP: General practitioner
vs: versus
