# Supplementary material for: Does children’s healthcare seeking change after participation in a musculoskeletal study? A register-based study
Source: BMC Prim Care. 2023 Dec 13;24:271. doi: 10.1186/s12875-023-02233-z (PMC10717731; doi:10.1186/s12875-023-02233-z)
Supplement: Supplementary file 1 — Additional file 1. [file 12875_2023_2233_MOESM1_ESM.docx]

**S1 List of ICD-10 diagnostic codes used for exclusion of children with a chronic or severe disease.**

This list only includes diagnostic codes identified in this studies population and is not a full/ exhaustive list of ICD-10 diagnostic codes identifying chronic and severe diseases in young children. In the Danish National Patient Register all ICD-10 codes begin with a D followed by the ICD-10 code.

| DF501 | **Atypical anorexia nervosa** |
| --- | --- |
| DF508 | **Other eating disorders** |
| DF509 | **Eating disorder, unspecified** |
| DF800 | **Specific speech articulation disorder** |
| DF808 | **Other developmental disorders of speech and language** |
| DQ010 | **Frontal encephalocele** |
| DQ011 | **Nasofrontal encephalocele** |
| DQ012 | **Occipital encephalocele** |
| DQ030 | **Malformations of aqueduct of Sylvius** |
| DQ031 | **Atresia of foramina of Magendie and Luschka** |
| DQ038 | **Other congenital hydrocephalus** |
| DQ039 | **Congenital hydrocephalus, unspecified** |
| DQ042 | **Holoprosencephaly** |
| DQ043 | **Other reduction deformities of brain** |
| DQ044 | **Septo-optic dysplasia** |
| DQ045 | **Megalencephaly** |
| DQ051 | **Thoracic spina bifida with hydrocephalus** |
| DQ053 | **Sacral spina bifida with hydrocephalus** |
| DQ054 | **Unspecified spina bifida with hydrocephalus** |
| DQ057 | **Lumbar spina bifida without hydrocephalus** |
| DQ058 | **Sacral spina bifida without hydrocephalus** |
| DQ059 | **Spina bifida, unspecified** |
| DQ061 | **Hypoplasia and dysplasia of spinal cord** |
| DQ063 | **Other congenital cauda equina malformations** |
| DQ068 | **Other specified congenital malformations of spinal cord** |
| DQ201 | **Double outlet right ventricle** |
| DQ203 | **Discordant ventriculoarterial connection** |
| DQ204 | **Double inlet ventricle** |
| DQ206 | **Isomerism of atrial appendages** |
| DQ208 | **Other congenital malformations of cardiac chambers and connections** |
| DQ209 | **Congenital malformation of cardiac chambers and connections, unspecified** |
| DQ210 | **Ventricular septal defect** |
| DQ211 | **Atrial septal defect** |
| DQ212 | **Atrioventricular septal defect** |
| DQ213 | **Tetralogy of Fallot** |
| DQ214 | **Aortopulmonary septal defect** |
| DQ220 | **Pulmonary valve atresia** |
| DQ221 | **Congenital pulmonary valve stenosis** |
| DQ222 | **Congenital pulmonary valve insufficiency** |
| DQ223 | **Other congenital malformations of pulmonary valve** |
| DQ224 | **Congenital tricuspid stenosis** |
| DQ225 | **Ebstein anomaly** |
| DQ228 | **Other congenital malformations of tricuspid valve** |
| DQ229 | **Congenital malformation of tricuspid valve, unspecified** |
| DQ230 | **Congenital stenosis of aortic valve** |
| DQ232 | **Congenital mitral stenosis** |
| DQ233 | **Congenital mitral insufficiency** |
| DQ234 | **Hypoplastic left heart syndrome** |
| DQ238 | **Other congenital malformations of aortic and mitral valves** |
| DQ240 | **Dextrocardia** |
| DQ249 | **Congenital malformation of heart, unspecified** |
| DQ250 | **Patent ductus arteriosus** |
| DQ251 | **Coarctation of aorta** |
| DQ254 | **Other congenital malformations of aorta** |
| DQ255 | **Atresia of pulmonary artery** |
| DQ256 | **Stenosis of pulmonary artery** |
| DQ258 | **Other congenital malformations of great arteries** |
| DQ259 | **Congenital malformation of great arteries, unspecified** |
| DQ261 | **Persistent left superior vena cava** |
| DQ262 | **Total anomalous pulmonary venous connection** |
| DQ263 | **Partial anomalous pulmonary venous connection** |
| DQ264 | **Anomalous pulmonary venous connection, unspecified** |
| DQ603 | **Renal hypoplasia, unilateral** |
| DQ614 | **Renal dysplasia** |
| DQ620 | **Congenital hydronephrosis** |
| DQ622 | **Congenital megaloureter** |
| DQ623 | **Other obstructive defects of renal pelvis and ureter** |
| DQ627 | **Congenital vesico-uretero-renal reflux** |
| DQ628 | **Other congenital malformations of ureter** |
| DQ631 | **Lobulated, fused and horseshoe kidney** |
| DQ639 | **Congenital malformation of kidney, unspecified** |
| DQ640 | **Epispadias** |
| DQ642 | **Congenital posterior urethral valves** |
| DQ643 | **Other atresia and stenosis of urethra and bladder neck** |
| DQ644 | **Malformation of urachus** |
| DQ647 | **Other congenital malformations of bladder and urethra** |
| DQ648 | **Other specified congenital malformations of urinary system** |
| DQ722 | **Congenital absence of both lower leg and foot** |
| DQ723 | **Congenital absence of foot and toe(s)** |
| DQ743 | **Arthrogryposis multiplex congenita** |
| DQ796 | **Ehlers-Danlos syndrome** |
| DQ822 | **Mastocytosis** |
| DQ824 | **Ectodermal dysplasia (anhidrotic)** |
| DQ860 | **Fetal alcohol syndrome (dysmorphic)** |
| DQ868 | **Other congenital malformation syndromes due to known exogenous causes** |
| DQ897 | **Multiple congenital malformations, not elsewhere classified** |

**S2 List of Service codes (incl. Danish description) used for estimating number of contacts with Physiotherapists, Chiropractors, and General Practitioners.**

| **Physiotherapy** | | **Chiropractic** | |
| --- | --- | --- | --- |
| \| 510101 \| \| --- \| \| 510102 \| \| 510103 \| \| 510104 \| \| 510105 \| \| 510110 \| \| 510111 \| \| 510112 \| \| 510113 \| \| 510120 \| \| 510121 \| \| 510122 \| \| 510201 \| \| 510202 \| \| 513201 \| \| 513301 \| \| 514001 \| \| 514501 \| \| 514511 \| \| 515101 \| \| 515102 \| \| 518050 \| \| \| 570110 \| \| --- \| \| 570115 \| \| 570311 \| \| 570312 \| \| 570313 \| \| 570314 \| \| 570315 \| \| 570321 \| \| 570322 \| \| 570323 \| \| 570324 \| \| 570325 \| \| 570421 \| \| 573201 \|  \| 610101 \| \| --- \| \| 610104 \| \| 610106 \| \| 614006 \| \| 620101 \| \| 620102 \| \| 620103 \| \| 620104 \| \| 620105 \| \| 620110 \| \| 620111 \| \| 620112 \| \| 620113 \| \| 620115 \| \| 620120 \| \| 620121 \| \| 620122 \| \| 620125 \| \| 620201 \| \| 623201 \| \| 623301 \| \| 624001 \| \| 624002 \| \| 624003 \| \| 624004 \| \| 624005 \| \| 624006 \| \| 624007 \| \| 624008 \| \| 624009 \| \| 624101 \| \| 624102 \| \| 624103 \| \| 624104 \| \| 624105 \| \| 624106 \| \| 624107 \| \| 624108 \| \| 625001 \| \| 625002 \| \| 625003 \| \| 625004 \| \| 625005 \| \| 625006 \| \| 625007 \| \| 625008 \| \| 625101 \| \| 625102 \| \| 625103 \| \| 625104 \| \| 625105 \| \| 625106 \| \| 625107 \| \| 625108 \| \| 626001 \| \| 626002 \| \| 626003 \| \| 626004 \| \| 626005 \| \| 626006 \| \| 626007 \| \| 626008 \| \| 626101 \| \| 626102 \| \| 626103 \| \| 626104 \| \| 626105 \| \| 626106 \| \| 626107 \| \| 626108 \| \| 627001 \| \| 627002 \| \| 627003 \| \| 627004 \| \| 627005 \| \| 627006 \| \| 627007 \| \| 627008 \| \| 627101 \| \| 627102 \| \| 627103 \| \| 627104 \| \| 627105 \| \| 627106 \| \| 627107 \| \| 627108   \| 650110 \| \| --- \| \| 650115 \| \| 650311 \| \| 650312 \| \| 650313 \| \| 650314 \| \| 650315 \| \| 650421 \| \| 653201 \| \| 653301 \| \| \| | \| Øvelsesterapi *indv* \| \| --- \| \| Afspænding *indv* \| \| Massage \| \| Termiske behandling \| \| El-stimulation \| \| Første konsultation \| \| Normal behandling \| \| Kort behandling \| \| Opflg. træningstera. \| \| Første konsultation i forbindelse med holdtræning \| \| Normalbehandling i forbindelse med holdtræning \| \| Kort behandling i forbindelse med holdtræning \| \| Øvelsesterapi *hold* \| \| Afspænding *hold* \| \| Telefonisk henvendelse \| \| Deltagelse i møde \| \| Holdundervisning \| \| Holdundervisning s51 \| \| 514511 findes ikke i takstmappen \| \| Forundersøgelse A65 \| \| Holdbehandling A65 \| \| Holdtræning(spec.51) \|  \| 1. kons \| \| --- \| \| Statuskonsultation \| \| Fys.hon.½ tim. 1.prs \| \| Fys.hon.½ tim.2prs. \| \| fys.hon.½ tim. 3 prs \| \| Fys.hon. ½ tim. 4prs \| \| Fys.hon. ½ tim. 5 pr \| \| rideskole hon. 1 prs \| \| Rideskole hon. 2 prs \| \| rideskole hon. 3 prs \| \| Rideskole hon. 4.prs \| \| Rideskole hon. 5.prs \| \| Rideklub m.hal 1 pr \| \| Telefonisk henvendelse \|  \| Øvelsesterapi *indv* \| \| --- \| \| Termiske behandling \| \| Ultralyd \| \| Holdtr. 6 per. egne \| \| Øvelsesterapi*indv* \| \| \| Afspænding *indv* \| \| \| Massage \| \| \| Termiske behandling \| \| \| El-stimulation \| \| \| Første konsultation \| \| \| Normal behandling \| \| \| Kort behandling \| \| \| Opflg. træn.terapi. \| \| \| Statuskonsultation \| \| \| Første konsultation i forbindelse med holdtræning \| \| \| Normal behandling i forbindelse med holdtræning \| \| \| Kort behandling i forbindelse med holdtræning \| \| \| Statuskonsultation på samme dag som hold \| \| \| Øvelsesterapi *hold* \| \| \| Telefonisk henvendelse \| \| \| Deltagelse i møde \| \| \| Holdtr. 1 per. egne \| \| \| Holdtr. 2 per. egne \| \| \| Holdtr. 3 per. egne \| \| \| Holdtr. 4 per. egne \| \| \| Holdtr. 5 per. egne \| \| \| Holdtr. 6 per. egne \| \| \| Holdtr. 7 per. egne \| \| \| Holdtr. 8 per. egne \| \| \| Holdtr. 9 per. egne \| \| \| Holdtræn. 1 person \| \| \| Holdtræn. 2 personer \| \| \| Holdtræn. 3 personer \| \| \| Holdtræn. 4 personer \| \| \| Holdtræn. 5 personer \| \| \| Holdtræn. 6 personer \| \| \| Holdtræn. 7 personer \| \| \| Holdtræn. 8 personer \| \| \| Bassint. 1 per. egne \| \| \| Bassint. 2 per. egne \| \| \| Bassint. 3 per. egne \| \| \| Bassint. 4 per. egne \| \| \| Bassint. 5 per. egne \| \| \| Bassint. 6 per. egne \| \| \| Bassint. 7 per. egne \| \| \| Bassint. 8 per. egne \| \| \| Bassintr. 1 person \| \| \| Bassintr. 2 personer \| \| \| Bassintr. 3 personer \| \| \| Bassintr. 4 personer \| \| \| Bassintr. 5 personer \| \| \| Bassintr. 6 personer \| \| \| Bassintr. 7 personer \| \| \| Bassintr. 8 personer \| \| \| hold 1per.egne 51 62 \| \| \| Hold 2per.egne 51 62 \| \| \| Hold 3per.egne 51 62 \| \| \| Hold 4per.egne 51 62 \| \| \| Hold 5per.egne 51 62 \| \| \| Hold 6per.egne 51 62 \| \| \| hold 7per.egne 51 62 \| \| \| Hold 8per.egne 51 62 \| \| \| Hold 1.per.amt 51 62 \| \| \| Hold 2per.amt 51 62 \| \| \| Hold 3per.amt 51 62 \| \| \| Hold 4per.amt 51 62 \| \| \| Hold 5per.amt 51 62 \| \| \| Hold 6 per.amt 51 62 \| \| \| Hold 7per.amt 51 62 \| \| \| Hold 8per.amt 51 62 \| \| \| Bas. 1per.egne 51 62 \| \| \| Bas. 2per.egne 51 62 \| \| \| Bas. 3per.egne 51 62 \| \| \| Bas. 4per.egne 51 62 \| \| \| Bas 5per.egne 51 62 \| \| \| Bas. 6per.egne 51 62 \| \| \| Bas 7per.egne 51 62 \| \| \| Bas 8per.egne 51 62 \| \| \| Bas 1 per.amt. 51 62 \| \| \| Bas. 2per.amt 51 62 \| \| \| Bas. 3per.amt 51 62 \| \| \| Bas. 4per.amt 51 62 \| \| \| Bas. 5per.amt 51 62 \| \| \| Bas. 6per.amt 51 62 \| \| \| Bas. 7per.amt 51 62 \| \| \| Bas. 8per.amt 51 62 \| \|  \| 1. konsultation \| \| --- \| \| Statuskonsultation \| \| Fys.hon. ½ tim.1 prs \| \| Fys.hon. ½ tim.2 prs \| \| Fys.hon.½ tim. 3 prs \| \| Fys. non ½ tim. 4 pr \| \| Fys.hon. ½ tim 5 prs \| \| Rideklub m.hal 1 prs \| \| Telefonisk henvendelse \| \| Deltagelse i møde \| | \| 531010 \| \| --- \| \| 531011 \| \| 531012 \| \| 531015 \| \| 531016 \| \| 531017 \| \| 531020 \| \| 531021 \| \| 531022 \| \| 531030 \| \| 531031 \| \| 531032 \| \| 531033 \| \| 531034 \| \| 531035 \| \| 531036 \| \| 531037 \| \| 533011 \| \| 533012 \| \| \| 641010 \| \| --- \| \| 641011 \| \| 641012 \| \| 641020 \| \| 641021 \| \| 641022 \| \| 641030 \| \| 641031 \| \| 641032 \| \| 641033 \| \| 641034 \| \| 641040 \| \| 641041 \| \| 641042 \| \| 641043 \| \| 641044 \| \| 641050 \| \| 641051 \| \| 641052 \| \| 641053 \| \| 641054 \| \| 641060 \| \| 641061 \| \| 641062 \| \| 641063 \| \| 641065 \| \| 643011 \| \| 643012 \| \| | \| Kir. kli. grundyd. a \| \| --- \| \| kombi 1010/3001 \| \| kombi 1010/3002 \| \| Forundersøgelse af patient, der ikke har været i k \| \| Forundersøgelse af patient, der har været i klinik \| \| Forundersøgelse af patient, der har været i klinik \| \| Kir. kli. grundyd. b \| \| kombi 1020/3001 \| \| kombi 1020/3002 \| \| Kir. klinisk ydelse \| \| kombi 1030/3001 \| \| kombi 1030/3002 \| \| kombi 1030/3003 \| \| kombi 1030/3004 \| \| Afgrænset opfølgende konsultation \| \| Almindelig opfølgende konsultation \| \| Udvidet opfølgende konsultation \| \| intr. til superviseret træning \| \| superviseret træning \|  \| Kir. kli. grundyd. a \| \| --- \| \| kombi 1010/3001 \| \| kombi 1010/3002 \| \| Kir. kli. grundyd. b \| \| kombi 1020/3001 \| \| kombi 1020/3002 \| \| Kir. klinisk ydelse \| \| kombi 1030/3001 \| \| kombi 1030/3002 \| \| kombi 1030/3003 \| \| kombi 1030/3004 \| \| Kons Kronik \| \| Kons Kronik \| \| Kons Kronik \| \| Kons Kronik \| \| Kons Kronik \| \| Forundersøgelse vedr. cervikal diskusprolaps \| \| Forundersøgelse af patienter i aktuelt behandlings \| \| Opfølgende konsultation efter 2 uger \| \| Opfølgende konsultation efter 4 uger \| \| Afsluttende forløbskonsultation efter 8 uger \| \| Forundersøgelse vedr. spinalstenose \| \| Forundersøgelse af patienter i aktuel behandlingsf \| \| opfølgende konsultation efter 4 uger \| \| Afsluttende konsultation efter 3 måneder \| \| Opfølgende konsultation \| \| intr. til superviseret træning \| \| superviseret træning \| |

| **General Practice** | | **General Practice** | |
| --- | --- | --- | --- |
| \| 800101 \| \| --- \| \| 800102 \| \| 800103 \| \| 800104 \| \| 800105 \| \| 800106 \| \| 800107 \| \| 800108 \| \| 800109 \| \| 800110 \| \| 800120 \| \| 800121 \| \| 800122 \| \| 800201 \| \| 800411 \| \| 800421 \| \| 800431 \| \| 800441 \| \| 800451 \| \| 800461 \| \| 800471 \| \| 800491 \| \| 800501 \| \| 800602 \| \| 802305 \| \| 803201 \| \| 803301 \| \| 803302 \| \| 803303 \| \| 803304 \| \| 804001 \| \| 804002 \| \| 804003 \| \| 804006 \| \| 804013 \| \| 804021 \| \| 804022 \| \| 804029 \| \| 804044 \| \| 804046 \| \| 804050 \| \| 804055 \|  \| 804056 \| \| --- \| \| 804063 \| \| 804072 \| \| 804076 \| \| 804088 \| \| 804105 \| \| 804106 \| \| 804117 \| \| 804126 \| \| 804128 \| \| 804129 \| \| 804130 \| \| 804135 \| \| 804139 \| \| 804140 \| \| 804142 \| \| 804144 \| \| 804145 \| \| 804146 \| \| 804153 \| \| 804165 \| \| 804166 \| \| 804171 \| \| 804172 \| \| 804173 \| \| 804176 \| \| 804183 \| \| 804184 \| \| 804198 \| \| 804200 \| \| 804213 \| \| 804214 \| \| 804215 \| \| 804230 \| \| 804249 \| \| 804250 \| \| 804251 \| \| 804256 \| \| 804257 \| \| 804280 \| \| 804282 \| \| 804283 \| \| 804284 \| \| 804286 \| \| 804292 \| \| 804293 \| | \| Konsultation \| \| \| --- \| --- \| \| Beh. 2.sikr.i sa.hj. \| \| \| Forebyggelseskons. \| \| \| F.kons iskæmisk hjer \| \| \| E-mail konsultation \| \| \| Aftalt forebygg.kons \| \| \| Forløbs.diabetes \| \| \| aft. diab.kontrol \| \| \| telefonkons. ved forløbsydelse diabetes \| \| \| e-komm. ved forløbsydelse diabetes \| \| \| Aftalt specifik forebyggende indsats \| \| \| Opsøgende hjemmebesøg \| \| \| Abortstøttesamtale \| \| \| Tlf.Kons. \| \| \| Besøg I \| \| \| Besøg II \| \| \| Besøg III \| \| \| Besøg IV \| \| \| Besøg V \| \| \| Besøg VI \| \| \| Besøg i vagttiden \| \| \| Besøg På Ruten \| \| \| Tlf.kons.u.bes./kon. \| \| \| Tlf.kons.m.bes./kon. \| \| \| Ops. hjemmebesøg \| \| \| Telefonkonsultation \| \| \| Møde 1 modul \| \| \| Møde 2 moduler \| \| \| Møde 3 moduler \| \| \| Møde udover 3 modul. \| \| \| Patientsamtaler \| \| \| Opsøgende virksomhed \| \| \| Samtaleterapi \| \| \| #2-aftale Århus ak. \| \| \| Udskrivningskonfer. \| \| \| Samtalebeh. 1 samt. \| \| \| Samtalebeh. 2 samt. \| \| \| Forebygkons diabetes \| \| \| §2A35Kons.h./besøg \| \| \| Ops. lægebesøg A35 \| \| \| Samtalebeh. 40 min. \| \| \| Tel. kons. A50 \| \| \|  \| \| \| Konsultation A50 \| \| \| Samtaleterapiforløb \| \| \| Længere enkeltkons. \| \| \| Konsultation \| \| \| Forebyg.helbreds.A60 \| \| \| HebB stofmis mæn sex \| \| \| Samtaleterapi B080 \| \| \| Aftalekons. B050 \| \| \| Årskontrol amt 50 \| \| \| Besøg 1 dags varigh. \| \| \| Besøg ½ ags varigh. \| \| \| Konference 4 moduler \| \| \| Demensudredning B080 \| \| \| Tlf. kons. 2 / B035 \| \| \| E-mail kons sende sv \| \| \| E-mail kons. besvar \| \| \| Terminal første kont \| \| \| besøg i hjemmet \| \| \| Støtte pårørende \| \| \| hjem.føds.Læsø R81 \| \| \| Motion/recept udredn \| \| \| 3mdr.kontrol kons \| \| \| hjemmebesøg R084 \| \| \| Hjemmebesøg \| \| \| Hjemmebesøg \| \| \| opf. hjemmebesøg R83 \| \| \| Besøg 4, aflastning \| \| \| Hjemmebesøg \| \| \| Hjemmebesøg \| \| \| Afst.tillæg \| \| \| Opf.hj-besøg 1. R082 \| \| \| Opf.hj-besøg 1. R082 \| \| \| Opf.hj.b. 2/3 R082 \| \| \| 1.kont. opf.hj.b.R85 \| \| \| Hjemmebesøg \| \| \| hjem.bes.udsk. R84 \| \| \| tlf.kons. R84 \| \| \| Hjemmebesøg \| \| \| udskr. besøg R081 \| \| \| REG H demens us \| \| \| REG H Årlig heldbredsundersøgelse \| \| \| REG H Sygebesøg \| \| Besøg/møder R84 \| \| Gladsaxe og Gentofte - årlig helbredsus for børn og unge under 18 år \| \| Kons Region Hovedstaden \| \| Tværsektoriel lægelig kommunikation i pt forløb \| | \| 804325 \| \| --- \| \| 804326 \| \| 804327 \| \| 804328 \| \| 804329 \| \| 804331 \| \| 804333 \| \| 804334 \| \| 804335 \| \| 804353 \| \| 804354 \| \| 804374 \| \| 804378 \| \| 804379 \| \| 804391 \| \| 804392 \| \| 804397 \| \| 804407 \| \| 804411 \| \| 804412 \| \| 804413 \| \| 804417 \| \| 804418 \| \| 804420 \| \| 804421 \| \| 804422 \| \| 804423 \| \| 804428 \| \| 804434 \| \| 804435 \| \| 804436 \| \| 804442 \| \| 804443 \| \| 804444 \| \| 804461 \| \| 804463 \| \| 804467 \| \| 804471 \| \| 804501 \| \| 804503 \| \| 804511 \| \| 804530 \| \| 804546 \| \| 804547 \| \| 804549 \|  \| 804609 \| \| --- \| \| 804610 \| \| 804612 \| \| 804613 \| \| 804615 \| \| 804642 \| \| 804657 \| \| 804658 \| \| 804659 \| \| 804669 \| \| 804670 \| \| 804676 \| \| 804810 \| \| 806101 \| \| 808110 \| \| 808120 \| \| 808130 \| \| 808140 \| \| 808141 \| \| 808142 \| \| 808150 \| \| 808210 \| \| 808211 \| \| 808212 \| \| 808213 \| \| 808214 \| \| 808215 \| \| 808216 \| \| 808217 \| \| 808701 \| \| 808702 \| \| 808708 \| \| 808910 \| \| 810101 \| \| 810102 \| \| 810471 \| \| 810501 \| \| 810602 \| \| 820101 \| \| 820102 \| \| 820471 \| \| 820501 \| \| 820602 \| | \| Kons.helbred R84 \| \| --- \| \| Kons.helbred R84 \| \| Hjemmebesøg \| \| Hjemmebesøg \| \| Hjemmebesøg \| \| Sundhedstjek R084 \| \| Care M. uge 1 R082 \| \| Care M. uge 14 R082 \| \| Eks.kons.CareM R082 \| \| Tlf kons DÆMP \| \| Tlf kons SAD r83 \| \| Delt. konf R83 \| \| Samtaleterapi \| \| Kons §2 aftale Region Sjælland \| \| Skema samtale R82 \| \| Kons 4392 - probl.løs.samt.R82 \| \| Telederma R81 \| \| 4407 - Helbr.samtale R082 \| \| Forb.helbr.us R083 \| \| fok.helbr.us. R83 \| \| Helbredssamt R83 \| \| Årskontr.diabetes \| \| Terminalpleje amt35 \| \| konsultation a42 \| \| tlf.kons.u.besøg a42 \| \| tlf.kons.m.besøg a42 \| \| besøg a42 \| \| Særlig kons.vagt.a42 \| \| kont.borger R083 \| \| Video.konf.R083 \| \| Video kons og projektkode \| \| Hjemmebesøg \| \| Hjemmebesøg \| \| Hjemmebesøg \| \| Hjemmebesøg \| \| Hjemmebesøg \| \| Demensudred. §2 A65 \| \| Besøg i vagttid R081 \| \| 1.besøg/hospitel A42 \| \| Frems. digit billed \| \| Kons.addition a65 \| \| Socialmed. samarb. \| \| Demensundersøge. A20 \| \| Demensunder. Kbh Kom \| \| Årskontrol,diab. a20 \| \|  \| \| Krisesamtale kritisk syge \| \| Samtale terapi kritisk syge \| \| Tilmelding T2D \| \| Tilmelding T2D \| \| Tilmelding T2D \| \| Særlig kons. vagttid \| \| Opfølg.samt. i kons. \| \| Opfølg.samt. i hjem \| \| udskrivningskonf.a60 \| \| Fremmøde ved udskriv. konf \| \| Hjemmebesøg \| \| Opfølgning efter indlæggelse \| \| Konsul V/hjælpeper \| \| Samtaleterapi \| \| 1.Undersøgelse \| \| 2.Undersøgelse \| \| 3.Undersøgelse \| \| 4.Undersøgelse \| \| Særkons. Sv.Forebyg. \| \| 4 Hj. Fødende 1.Uge \| \| Unders.v.vejl.9 Uger \| \| Undersøgelse \| \| 1.Undersøgelse \| \| 2.Undersøgelse \| \| 3.Undersøgelse \| \| 4.Undersøgelse \| \| 5.Undersøgelse \| \| 6.Undersøgelse \| \| 7.Undersøgelse \| \| Tillæg v. MFR-vacc. \| \| Tillæg v. Hib-vacc. \| \| Tillæg ved børnevaccinationer hvor der ikke er en \| \| 8910 - Influenzavacc. pens. \| \|  \| \| Konsultation \| \| Beh. 2.sikr.i sa.hj. \| \| Besøg \| \| Telf.kons.uden besøg \| \| Telf.kons.med besøg \| \| Konsultation \| \| Beh. 2.sikr.i sa.hj. \| \| Besøg i vagttiden \| \| Tlf.kons.u.bes./kon. \| \| Tlf.kons.m.bes./kon. \| |
